# The TGF-β pathway is activated by 5-fluorouracil treatment in drug resistant colorectal carcinoma cells

**DOI:** 10.18632/oncotarget.7895

**Published:** 2016-03-03

**Authors:** Gabriele Romano, Ludovica Santi, Maria Rosaria Bianco, Maria Rita Giuffrè, Mariateresa Pettinato, Cristina Bugarin, Cristina Garanzini, Leonilde Savarese, Silvia Leoni, Maria Grazia Cerrito, Biagio Eugenio Leone, Giuseppe Gaipa, Emanuela Grassilli, Michele Papa, Marialuisa Lavitrano, Roberto Giovannoni

**Affiliations:** ^1^ Department of Surgery and Translational Medicine, University of Milano-Bicocca, 20900, Monza, Italy; ^2^ Department of Surgery and Translational Medicine, University of Milano-Bicocca, c/o Department of Mental and Physical Health and Preventive Medicine, Second University of Naples, 80138, Naples, Italy; ^3^ M. Tettamanti Research Center, Pediatric Clinic, University of Milano Bicocca, 20900 Monza, Italy; ^4^ Laboratory of Neuronal Networks, Department of Mental and Physical Health and Preventive Medicine, Second University of Naples, 80138, Naples, Italy

**Keywords:** TGF-β, chemoresistance, 5-fluorouracil, colorectal cancer, SMAD3

## Abstract

TGF-β pathway is generally associated with the processes of metastasis, angiogenesis and EMT in cancer. Very little is known, however, about the role of TGF-β in cancer drug resistance. In this work, we show a specific activation of the TGF-β pathway in consequence of chemotherapeutic treatment in *in vivo* and *in vitro* models of colorectal carcinoma. 5-Fluorouracil (5FU) was able to stimulate the activation of SMAD3 and the transcription of specific genes such as *ACVRL1*, *FN1* and *TGFB1*. On the other hand, the specific inhibition of TGF-βRI was able to repress the 5FU-induced genes transcription and to restore the sensitivity of chemoresistant cells to the toxic action of the drug, by decreasing the expression of *BCL2L1* and *ID1* genes. The role of the TGF-β molecule in the chemoresistant colon carcinoma cells' response to 5FU was further demonstrated by conditioned medium (CM) experiments: CM from 5FU-treated chemoresistant cells was able to protect chemosensitive cells against the toxic action of 5FU. In conclusion, these findings showed the pivotal role of TGF-β pathway in colon cancer mechanisms of drug resistance suggesting new possible approaches in diagnosis and treatment of colon cancer patients.

## INTRODUCTION

TGF-β is known to have paradoxical roles in carcinogenesis and cancer development: in the early stages of oncogenesis TGF-β pathway activation is generally associated with oncosuppression [1], whereas in the more advanced stages of tumor development TGF-β promotes metastasis, angiogenesis, immunosuppression and Epithelial to Mesenchymal transition (EMT) [2, 3].

TGF-β ligands bind type 2 TGF-β receptor (TGF-βR2), which dimerize with an other TGF-βR2; the such formed dimer forms then a tetramer with a type 1 TGF-β receptor (TGF-βR1) dimer, causing TGF-βR1 phosphorylation [4]. TGF-βRI activates downstream pathways via the canonical SMAD pathway, in which receptor SMADs (SMAD2/3) form a complex with co-SMAD (such as SMAD4) and translocate to the nucleus to regulate transcription of target genes [5, 6]. In the non-canonical SMAD-independent pathways, TGF-β can activate or suppress a plethora of alternative signaling pathways, such as p38 mitogen-activated protein kinases (MAPK), phosphoinositide 3-kinase (PI3K)-AKT, Glycogen Synthase Kinase 3 (GSK3) and Rho GTPases. SMAD and non-SMAD pathways activation is strongly dependent on physiological or pathological cellular subset in which TGF-β exerts its action [7–9].

In epithelial tumors, TGF-β paradoxical role is due to the loss of the anti-proliferative effects of the TGF-β pathway in the first stages of tumor development and the consequent exacerbation of tumor-promoting effects [10]. For example, ATF3-mediated ID1 repression is one of the tumor suppressor arms of TGF-β signaling [11], but in patient-derived metastatic breast cancer cells TGF-β causes an aberrant increase of ID1 expression promoting lung metastasis [12].

In colon cancer models, TGF-β pathway hyper activation can eventually lead to the expression of PAI-1, and α-smooth muscle actin (α-SMA) in cancer associated fibroblasts (CAFs) [13] by creating a positive loop of TGF-β production and a tumor promoting microenvironment. TGF-β is also associated with tumor progression, neo-angiogenesis and lymph-node metastases in colorectal cancer, and it has been suggested as a possible biomarker for cancer progression and aggressiveness [14].

In addition, TGF-β is able to recruit macrophages to the tumor site and direct their response to a M2 phenotype, which is immunosuppressive and pro-angiogenetic [15, 16]. In spite of plenty of literature about the pivotal role of TGF-β in colon cancer, very little is known about the molecular mechanisms activated by TGF-β in colon cancer drug resistance. Recent findings showed that MED12 (Mediator Complex Subunit 12) expression was able to modulate TGF-β signaling and to influence chemotherapeutic response in colorectal cancer cells [17, 18]. However, this kind of induced resistance is generically associated to the acquisition of a mesenchymal phenotype by cancer cells, and not to a TGF-β-specific response to chemotherapeutic administration.

The aim of this work was to deepen the molecular aspects of TGF-β signaling in a colorectal cancer model of reversion of chemoresistance [19]. The translational relevance of this study is highlighted by the finding that TGF-β pathway was up-regulated in consequence of chemotherapeutic administration, and that specific inhibition of TGF-β signaling was able to restore drug sensitivity in colorectal cancer cells, in *in vivo* and *in vitro* models.

## RESULTS

### 5-fluorouracil treatment causes an activation of TGF-β pathway *in vivo*

**Figure 1:**
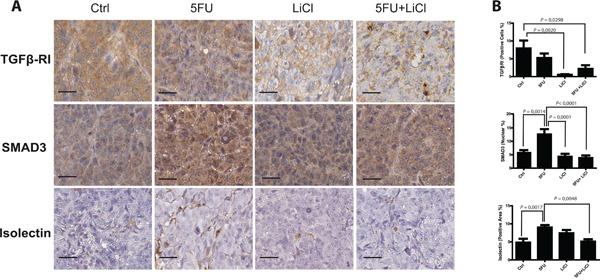
5-fluorouracil treatment causes an activation of TGF-β pathway in xenografted chemoresistant cells **A.** Representative images of IHC on HCT116p53KO tumor sections for each group of treatment. Bars represent 20 μm. 5FU increased SMAD3 nuclear translocation and tumor neo-angiogenesis. LiCl administration was able to downregulate TGF-βRI expression, to inhibit SMAD3 nuclear translocation and to restore basal levels of tumor vascularization. **B.** Quantification was performed on whole tumor sections excluding necrotic areas (8 sections per group of treatment). Significant *P*-values among groups after multiple comparisons are indicated above columns. Error bars represent SEM.

We previously set up a xenograft model of colon cancer chemoresistance reversion and in this model we reported that Lithium (LiCl), which inhibits glycogen synthase kinase 3 (GSK3), administration was able to re-sensitize chemoresistant colon cancer cells to 5-Fluorouracil (5FU) chemotherapeutic action [19]. Since it has been reported a relationship between GSK3 and TGF-β pathways in tumor progression of different carcinomas [20, 21] and it has been demonstrated the relationship between chemoresistance and TGF-β in colon cancer [17], we investigated how the TGF-β pathway could contribute to the chemoresistance/chemoreversion phenomenon in our model. Immunohistochemistry analysis of TGF-βRI on xenografted tumor masses indicated that LiCl administration caused a significant downregulation of this receptor kinase expression, independently from 5FU administration in chemoresistant xenograft tumors (Figure 1A and 1B, upper panels). On the other hand, 5FU treatment increased nuclear translocation of SMAD3 as compared to control group, whereas LiCl was able to restore basal levels of nuclear SMAD3 (Figure 1A and 1B, middle panels). As a result, LiCl-mediated TGF-βRI downregulation was able to contrast the 5FU-induced SMAD3 increased activation. Since the TGF-β pathway has a pro-angiogenic effect [22–24], we analyzed the microvasculature of the xenografted tumors in our model and we found a dramatic increase of tumor vascularization in consequence of 5FU administration (75mg/kg/d twice a week), whereas the combination of LiCl (160 mg/kg/d) and 5FU was able to significantly decrease the vasculature density, restoring the basal value (Figure 1A and 1B, bottom panels). Noteworthy, no significant changes in TGF-βRI expression, in SMAD3 localization nor in vascularization, were observed in chemosensitive HCT116 xenograft tumor sections (Supplementary Figure S1), suggesting that the observed molecular events are selective for chemoresistant tumors. Taken together, these findings indicated that LiCl and 5FU exert opposite effects on TGF-β signaling pathway and that such regulations are exclusive for chemoresistant cells.

### TGF-βRI inhibition reduces proliferation and increases cell death of chemoresistant cancer cells

In order to better characterize the molecular aspects of TGF-β pathway regulation by 5FU and LiCl, we established an *in vitro* 3D model of colon carcinoma cells. The 3D model consisted of a gelled extracellular matrix (ECM) bed, on which colorectal cancer cells were seeded at low density; cells were then cultured in a gradient of ECM and reduced serum condition (See Materials and Methods). The aim of this model was to reproduce, as much as possible, the tri-dimensional structure of an epithelial tumor.

**Figure 2:**
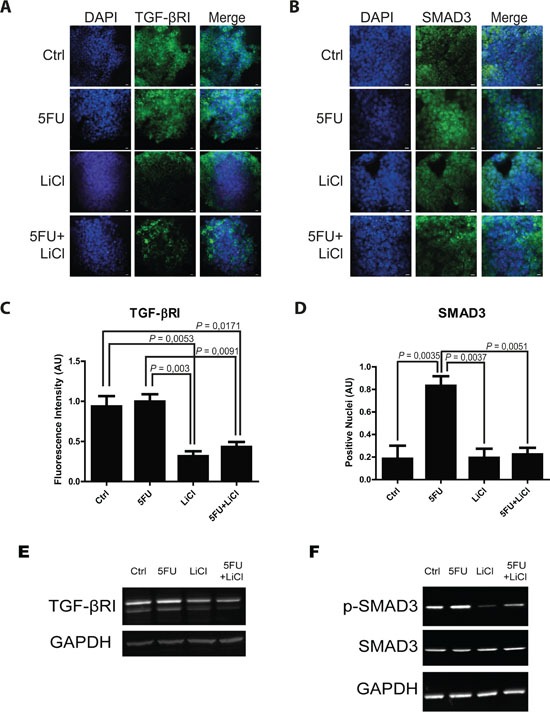
5-fluorouracil treatment causes an activation of TGF-β pathway in the *in vitro* 3D-cultured chemoresistant cells Representative pictures of immunofluorescence analysis in 3D-cultured chemoresistant cancer cells. Cells were immunostained for TGF-βRI **A.** or SMAD3 **B.** (green) and with DAPI (blue). Bars represent 20 μm. **C.** Lithium administration caused a reduction of TGF-βRI expression as compared to control group in immunofluorescence analysis. **D.** 5FU treatment increased SMAD3 nuclear translocation, whereas Lithium co-treatment with 5FU was able to restore the basal condition. Significant *P*-values among groups after multiple comparisons are indicated above columns. Error bars represent SEM. **E.** Western blotting analysis of TGF-βRI in 3D-cultured chemoresistant cancer cells showing a similar trend as in *in situ* analysis. **F.** Western blotting analysis of p-Ser204-SMAD3 and SMAD3 in 3D-cultured chemoresistant cancer cells showing an increase of activating phosphorylation on Serine 204 of SMAD3 upon 5FU treatment. Images in E and F are representative of at least three independent experiments. GAPDH was used as equal loading control. p-SMAD3, phospho serine 204 SMAD3.

**Figure 3:**
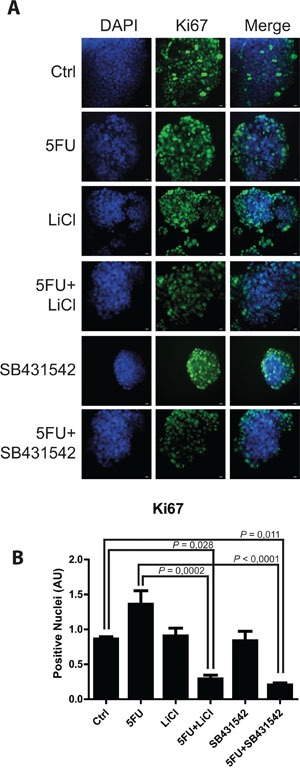
TGF-βRI inhibition reduced proliferation of 3D-cultured chemoresistant cancer cells **A.** Representative pictures of immunofluorescence analysis for Ki67 (marker of cell proliferation, green) on 3D-cultured HCT116p53KO chemoresistant cell lines. Bars represent 20 μm. Nuclei were stained with DAPI (blue). **B.** Lithium or SB431542 treatments in combination with 5FU strongly downregulated Ki67 expression. Significant *P*-values among groups after multiple comparisons are indicated above columns. Error bars represent SEM.

**Figure 4:**
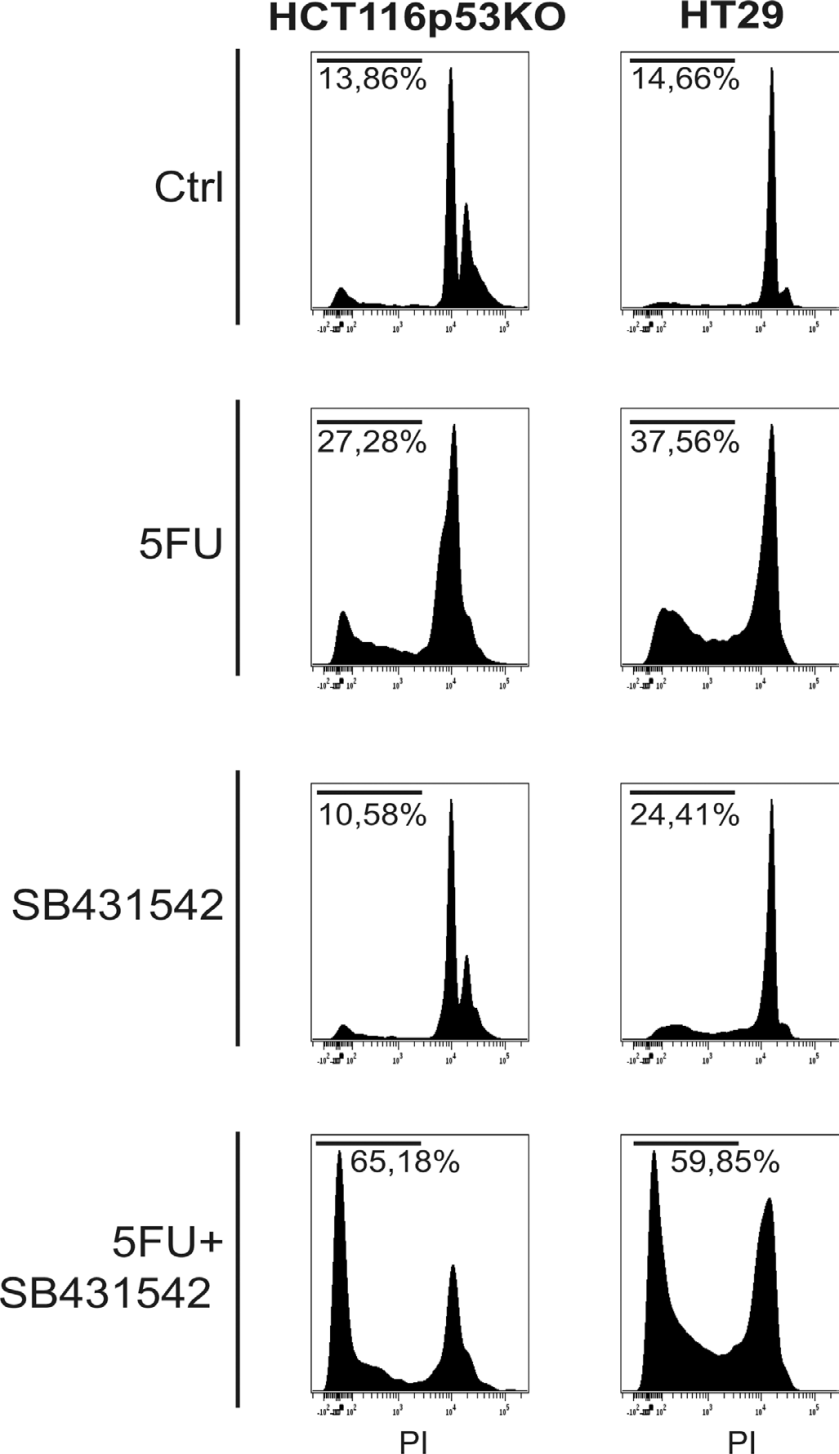
TGF-βRI inhibition increased cell death of 3D-cultured chemoresistant cancer cells Propidium iodide incorporation assay analysis on chemoresistant HT-29 and HCT116p53KO cells: TGF-βRI inhibition caused a re-sensitization to 5FU toxicity, dramatically increasing chemoresistant cell death (sub-G0/G1 cell populations indicated as percentage). All images are representative of at least three independent experiments.

Consistently to our findings in the *in vivo* model, immunofluorescence and immunoblot analysis of 3D cultured chemoresistant cells treated with 5FU, LiCl or a combination thereof revealed a downregulation of TGF-βRI exerted by LiCl (Figure 2A, 2C, 2E). Moreover, it was observed a strong SMAD3 nuclear translocation in consequence of 5FU treatment (Figure 2B and 2D) which was abolished when cells were co-treated with 5FU and LiCl. To further support these findings, immunoblot analysis for pSer204-SMAD3 was performed on HCT116p53KO cells. As shown in Figure 2F, 5FU administration caused a significant increase in SMAD3 phosphorylation, which was abolished by LiCl administration. No significant changes in SMAD3 nuclear translocation or TGF-βRI expression were detected in chemosensitive HCT116 cells (Supplementary Figure S2). The downstream activation of SMAD3 did not involve regulation of SMAD4, as expression levels of this protein did not change in any treatment nor in xenograft (Supplementary Figure S3A, S3B) nor in 3D-cultured tumor cells (Supplementary Figure S3C). On the basis of these results, we hypothesized an involvement of the TGF-βRI in the chemoresistant cells response to 5FU. In order to verify if the LiCl-mediated TGF-βRI downregulation was an off-target effect or a specific molecular regulation involved in chemoresistance, we inhibited the TGF-βRI by using SB431542, a well-known inhibitor of this serine/threonine kinase receptor [12, 25, 26]. Proliferation analysis showed that SB431542 treatment was able to dramatically decrease Ki67 expression in combination with 5FU, in HCT116p53KO cells (Figure 3). Furthermore, cell death analysis by the Propidium Iodide (PI) incorporation assay revealed that the co-treatment with 5FU and SB431542 was able to significantly increase the number of cells in sub G0/G1 cell cycle phase (apoptotic or dead cells) not only in HCT116p53KO but also in HT-29 cells, another chemoresistant colon cancer cell line (Figure 4). Taken together these data suggested that the TGF-βRI modulation is involved in the chemoresistance/chemoreversion phenomenon.

### 5FU modulates TGF-β target genes expression in chemoresistant colon carcinoma cells

**Figure 5:**
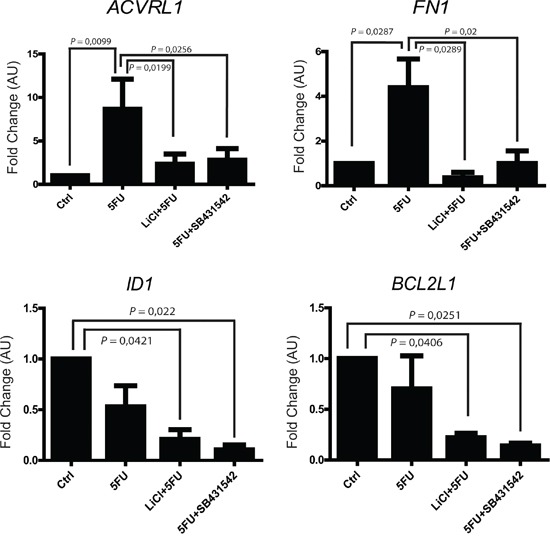
5FU modulates the mRNA expression of selected TGF-β target genes in chemoresistant colon carcinoma cells *ACVRL1* and *FN1* expression was upregulated by 5FU administration to chemoresistant cells. TGF-βRI inhibition (LiCl- or SB431542-mediated) in combination with 5FU treatment, not only reduced *ACVRL1* and *FN1* up-regulation, but also strongly repressed *ID1* and *BCL2L1* genes transcription. Columns represent the fold change values as compared to the control group, expressed by the 2^−ΔΔCt^ algorithm as detailed in Materials and Methods section. Significant *P*-values among groups after multiple comparisons are indicated above columns. Error bars represent SEM.

In order to better elucidate which TGF-β signaling pathway molecules are possibly involved in the colon cancer chemoresistance, we analyzed the expression levels of 84 different genes known to be fundamental players in TGF-β signaling. To this extent, HCT116p53KO 3D-cultured cells were treated with vehicle, 5FU, LiCl, SB431542 or a combination of 5FU and LiCl or 5FU and SB431542 and the corresponding RNA samples were analyzed by quantitative RT^2^-PCR Profiler Array (Qiagen). Expression array analysis revealed a strong up-regulation of TGF-β target genes in consequence of 5FU treatment: 52 genes were found to be up-regulated, 10 didn't reveal any change and 12 genes were downregulated (Supplementary Figure S4). Among the analyzed genes, 4 were selected for their significant modulation and biological function and further validated (Table 1): *ACVRL1* (Activin A receptor type II-like 1), known to be involved in angiogenesis and tumor growth; *FN1* (Fibronectin-1), a master regulator of ECM remodeling and cell-matrix adhesion; *ID1* (Inhibitor of DNA binding 1), which promotes cells proliferation and migration; *BCL2L1* (BCL-2 like 1), encoding for a well known anti-apoptotic protein. *ACRVL1* and *FN1* were found to be significantly upregulated by 5FU administration, whereas LiCl or SB431542 treatments inhibited such increased gene transcription (Figure 5). On the other hand, *ID1* and *BCL2L1* did not show any significant change in cells treated with 5FU alone, whereas LiCl or SB431542 co-treatments with 5FU strongly downregulated the expression of these two genes (Figure 5). Consistently with all previous data, LiCl and SB431542 had similar effects on gene expression profiling in chemoresistant cells. Taken together, these results suggested that, in chemoresistant colon carcinoma cells exposed to 5FU, the chemotherapeutic agent stimulates proliferative and pro-migratory signaling, whereas 5FU and LiCl or SB431542 co-treatments were able to abolish the 5FU-activated pathway. Furthermore, the 5FU and LiCl or SB431542 co-treatments were also able to inhibit the pro-survival signals in chemoresistant cells, thus re-sensitizing HCT116p53KO cells to the chemotherapeutic action.

**Table 1:** Expression of relevant TGFβ target genes in HCT116p53KO cells treated with 5FU alone and in combination with LiCl or SB431542

Gene	5FU vs Ctrl	*p*	5FU+LiCl vs 5FU	*p*	5FU+SB431542 vs 5FU	*p*
*ACVRL1*	7,99	<0.00001	−2,82	0,089853	−2,01	0,067599
*BCL2L1*	−2,00	0,118334	−2,81	0,043971	−3,99	0,003379
*FN1*	5,67	0,006394	1,00	0,675747	−2,83	0,034300
*ID1*	−2,00	0,310162	−2,81	0,000036	−4,01	0,036798

Data are expressed as fold change as compared to the indicated controls

### TGF-β1 exerts a protective action against 5-fluorouracil treatment

**Figure 6:**
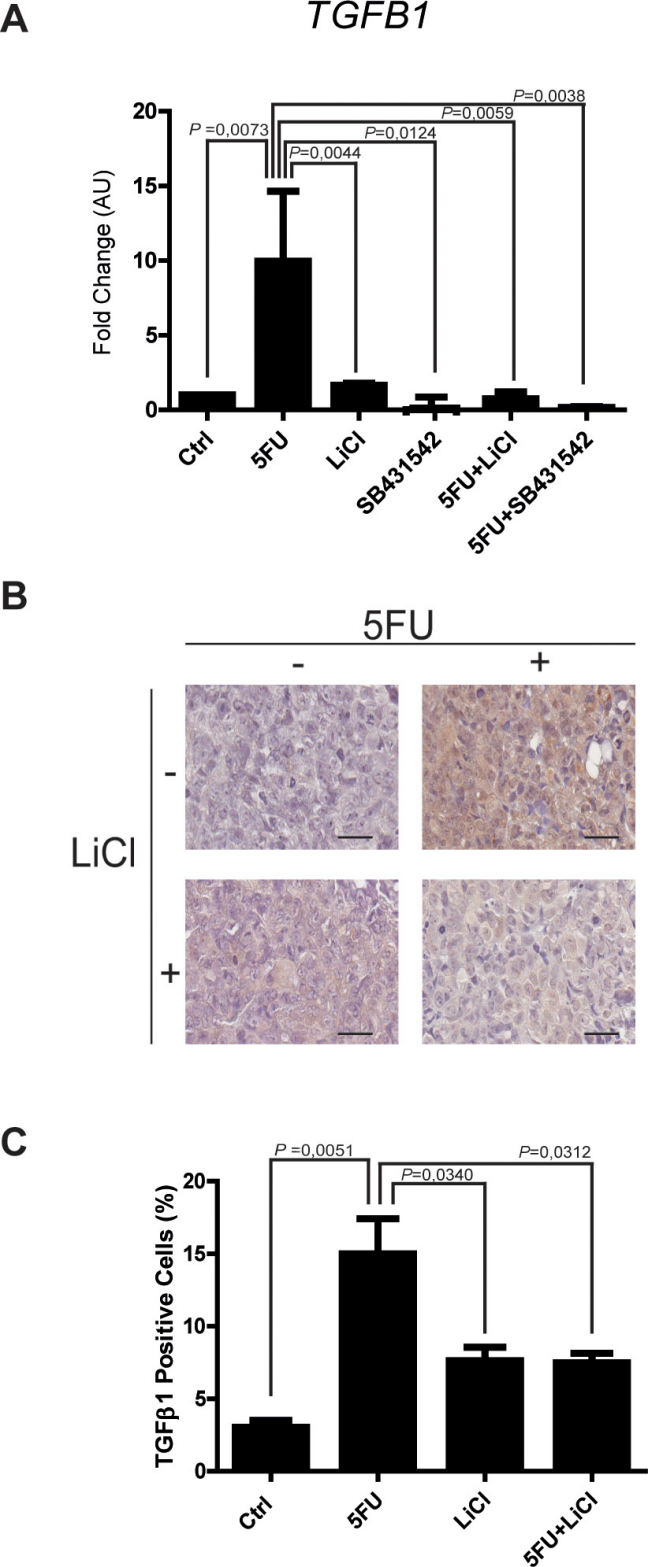
Expression of TGF-β1 is up-regulated by 5FU *in vitro* and *in vivo* **A.** Real time PCR analyses on mRNA extracted from chemoresistant HCT116p53KO cells treated with 5FU alone or in combination with LiCl or SB431542. The treatment with 5FU significantly up-regulated TGF-β1 expression, but this increase was abolished by the co-treatment with LiCl or SB431542. Columns represent the fold change values as compared to the control group, expressed by the 2^−ΔΔCt^ algorithm as detailed in Materials and Methods section. Significant *P*-values among groups after multiple comparisons are indicated above columns. Error bars represent SEM. **B.** Representative images of IHC on HCT116p53KO tumor sections for each group of treatment. Bars represent 20 μm. 5FU treatment increased TGF-β1 expression. LiCl administration was able to restore the expression of the cytokine to basal levels. **C.** Quantification was performed on whole tumor sections excluding necrotic areas (8 sections per group of treatment). Significant *P*-values among groups after multiple comparisons are indicated above columns. Error bars represent SEM.

**Figure 7:**
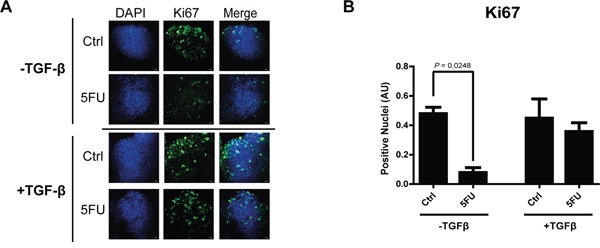
TGF-β1 exerts a protective role action against 5FU treatment in chemosensitive colon carcinoma cells **A.** Representative pictures of immunofluorescence analysis of Ki67 (green) on chemosensitive HCT116 carcinoma cells treated with 5FU with or without exogenous TGF-β1. Nuclei were stained with DAPI (blue). Bars represent 20 μm. **B.** TGF-β1 treatment protected HCT116 cells against 5FU toxicity, in terms of Ki67 expression (proliferation index). Significant *P*-values among groups after multiple comparisons are indicated above columns. Error bars represent SEM.

**Figure 8:**
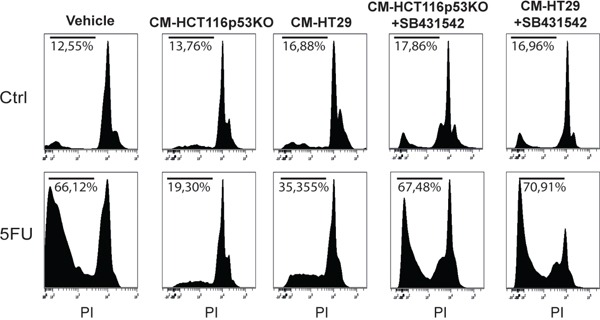
Conditioned medium from chemoresistant cells was able to protect chemosensitive HCT116 cells against 5FU toxicity Propidium iodide incorporation assay analysis of conditioned medium (CM) experiments. CM from HCT116p53KO or HT-29 cells that received 5FU administration was able to induce chemoresistance in HCT116 cells (sub-G0/G1 cell populations indicated as percentage). When the inhibitor of TGF-βRI, SB431542, was added to 5FU treatment, the conditioned medium from chemoresistant cells completely lost its protective action. All images are representative of at least three independent experiments.

The observation of increased activation of SMAD3 and the strong modulation of TGF-β target genes in consequence of 5FU treatment in chemoresistant colon carcinoma cells, led us to hypothesize a possible role of the TGF-β molecule in the cell response to 5FU. To verify this hypothesis, we investigated the expression levels of TGF-β1 in HCT116p53KO cells. Interestingly, we found that TGF-β1 expression was significantly increased by 5FU treatment and, oppositely, LiCl or SB431542 co-treatments with 5FU were able to restore basal levels of TGF-β1 (Figure 6A). Moreover, IHC analysis for TGF-β1 on HCT116p53KO xenografted tumors, revealed an increased protein expression in consequence of 5FU administration. Once more, LiCl administration was able to impair TGF-β1 increased levels (Figure 6B, 6C). This finding led us to speculate a kind of autocrine signal operated by chemoresistant cells in presence of 5FU. We then investigated if TGF-β1 could be a protective factor against 5FU toxicity. To this extent, we treated chemosensitive colon carcinoma HCT116 cells with TGF-β1 in combination with standard 5FU treatment and cell proliferation and death were measured. HCT116 cells, treated with both TGF-β1 and 5FU, did not show any decrease in Ki67 expression as compared to the 5FU-treated cells without TGF-β1 treatment (Figure 7A, 7B). These findings, consistently with previous observations, suggested that TGF-β1 treatment was able to protect chemosensitive cells against the action of 5FU. To further support the hypothesis of an autocrine protective loop of TGF-β1, we administered 5FU to HT-29 and HCT116p53KO cells, and then we added such conditioned medium to 5FU-treated chemosensitive cells. As shown in Figure 8, the medium conditioned from chemoresistant HT-29 or HCT116p53KO cells was able to protect chemosensitive cells from the chemotherapeutic toxicity. Moreover, if SB431542 and conditioned medium from chemoresistant cells were simultaneously added to chemosensitive cells, the protective effect of conditioned medium was impaired, and chemosensitivity restored (Figure 8).

## DISCUSSION

The results presented in this work showed that the TGF-β pathway has a pivotal role in colon cancer models of chemoresistance. TGF-β is known to be a main player in the processes of tumor development, metastasis and angiogenesis [1–3, 14, 27]; the TGF-β pathway has been recently associated to drug resistance [17], but very little is known about TGF-β pathway specific activation in the context of chemotherapeutic administration in colon carcinoma. We recently reported that LiCl, which inhibits GSK3, administration was able to re-sensitize chemoresistant colon cancer cells to 5FU chemotherapeutic action, *in vitro* and *in vivo* [19] and we used this model of chemoresistance to investigate a possible involvement of TGF-β pathway in this phenomenon. We observed that LiCl administration to xenografted tumors-bearing mice significantly downregulated TGF-βRI expression in chemoresistant tumors (Figure 1). A non-significant reduction was also observed in 5FU treated tumors that was not confirmed by any other findings and was probably due to the intrinsic variability of the xenograft model. SMAD3 is one of the main effectors downstream the TGF-βRI activation and the immunohistochemistry analysis of this marker surprisingly revealed that 5FU dramatically increased SMAD3 nuclear translocation, suggesting an activation of the TGF-β pathway (Figure 1). The treatment with LiCl abolished this induction, preventing SMAD3 to enter the nucleus even if the 5FU had been administered together with LiCl (Figure 1), suggesting that the inhibition of GSK3 pathway could switch off the TGF-β pathway activation induced by 5FU. Moreover, the LiCl-induced impairment of SMAD3 nuclear translocation was consistent with TGF-βRI downregulation observed in tumors from LiCl-treated mice. As TGF-β is known to be a main player in the neo-angiogenesis and vascular sprouting processes [22–24], we investigated if the observed modulation of TGF-βRI and SMAD3 molecules correlated with modifications in the microvasculature of the xenografted tumors. Again, consistently with a hypothesis of a specific modulation of TGF-β pathway induced by 5FU and LiCl treatments, we observed that the group of tumors that received 5FU showed increased vascularization as compared to control group (Figure 1). Furthermore, the number of microvessels was restored to basal levels by LiCl administration. Noteworthy, no significant changes were observed in chemosensitive tumors in SMAD3, TGF-βRI or vasculature modulation (Supplementary Figure S1).

In order to deepen the aspects of the involvement of TGF-β pathway in the chemoresistance and in the reversion of this phenotype, we investigated, in an *in vitro* model, the molecular events occurring in colon carcinoma cells exposed to 5FU. To better recapitulate the tumor tissue architecture and some microenvironment features *in vitro*, we used an established 3D-model [28, 29] mimicking a three-dimensional structure of a tissue. In this model, the down-regulation of TGF-βRI in LiCl-treated cells was confirmed (Figure 2A, 2C, 2E). The 5FU-induced SMAD3 nuclear translocation, as well as its inhibition due to LiCl treatment, was observed through immunofluorescence and immunoblot analyses (Figure 2B, 2D, 2F). Since TGF-β pathway activation induces Ser204-SMAD3 phosphorylation, in TGF-βRI-dependent manner [30], we analyzed the p-Ser204-SMAD3 levels in 3D-cultured cells and found an activation consistent with the nuclear translocation of SMAD3 (Figure 2F). Noteworthy, it has been reported that LiCl treatment was able to inhibit pSer204-SMAD3 levels [30] and SMAD3/SMAD4 transactivation via the cAMP– protein kinase A (PKA), AKT–GSK3β, and CRE-dependent signaling pathways [31]. No previous literature reports LiCl-mediated TGF-βRI downregulation. In order to exclude a LiCl off-target effect on TGF-βRI, we specifically inhibited TGF-βRI by using SB431542, a well-known TGF-βRI inhibitor [12, 25, 26]. We observed that TGF-βRI specific inhibition in combination with 5FU was able to re-sensitize chemoresistant cells to chemotherapeutic action, in terms of inhibition of proliferation (Figure 3) and induction of cell death (Figure 4). These findings excluded the possibility of an off-target effect of LiCl on TGF-βRI, and confirmed the active involvement of the TGF-β pathway in the phenomenon of chemoresistance.

Trying to unravel which molecules could be involved in the TGF-β pathway modulation in colon cancer chemoresistance, we performed a gene profiling by analyzing 84 TGF-β target genes: chemoresistant colon carcinoma cells that received 5FU showed an up-regulation of 52 out of 84 genes analyzed (Supplementary Figure S4). More interestingly, SB431542 and LiCl treatments were both able to inhibit 5FU-induced TGF-β target genes expression profiles. Among the analyzed genes, four were selected on the basis of significance and biological function and were further validated. *ACRVL1*, a receptor known to have role in neo-angiogenesis [32, 33] and tumor cell proliferation [34, 35] was found to be up-regulated by 5FU administration, while its expression was reduced in cells co-treated with 5FU with LiCl or SB431542 (Figure 5). 5FU alone also strongly induced *FN1*, a protein involved in ECM reorganization and cell to matrix adhesion processes [36, 37], but *FN1* up-regulation was abolished by the co-treatment of 5FU and LiCl or 5FU and SB431542 (Figure 5). The expression levels of *BCL2L1* (a gene encoding for anti-apoptotic proteins [38, 39]) were found to be consistent with cell death evaluated by PI incorporation assay (Figure 4): as expected, in chemoresistant cells, 5FU treatment did not induce any significant change in expression of an anti-apoptotic gene (Figure 5). On the other hand, the expression of *BCL2L1* was significantly reduced in cells co-treated with 5FU and LiCl or SB432541 (Figure 5). Interestingly, the inhibition of TGF-βRI (LiCl- or SB431542-mediated) in combination with 5FU, was able to significantly repress the expression of *ID1* (Figure 5), which is a strong promoter of TGF-β-mediated cell proliferation and migration [40–42].

Taken together, these findings suggested that the activation of TGF-β pathway was specifically induced by 5FU treatment in chemoresistant colon carcinoma cells, and that the target genes modulated during this phenomenon are involved in the regulation of surrounding microenvironment as well as in cell mechanisms of death and proliferation. In addition, the inhibition of TGF-β pathway not only was able to counteract 5FU-induced genes modulation, but it restored some of the mechanisms generally lost in chemoresistant cells (BCL2L1 and ID1).

The increase in nuclear localization of SMAD3 in our *in vivo* and *in vitro* models of chemoresistance together with the modulation of TGF-β target genes only in chemoresistant, 5FU-treated, colon carcinoma cells led us to hypothesize a possible role of the TGF-β molecule in the response to 5FU. TGF-β1 was found to be upregulated by chemotherapeutic treatment and restored to basal levels by co-treatment of 5FU with LiCl or SB431542 in HCT116p53KO cells *in vitro* and *ex vivo* (Figure 6). This finding suggested a sort of autocrine loop: the chemoresistant cells increase SMAD3 signaling, which increases TGF-β1 expression, which in turn increases SMAD3 signaling again. In fact, TGF-β1 is known to be the main inducer of TGF-β1 itself [43, 44]. In our model, the loop would be interrupted by TGF-βRI inhibition, as it is the receptor which is up-stream of the whole signaling pathway.

To support this hypothesis, we then investigated if TGF-β1 could be a protective factor against 5FU toxicity in chemosensitive colon carcinoma cells, HCT116. As expected, we observed that TGF-β1 treatment was able to protect HCT116 (chemosensitive) cells to the action of 5FU (Figure 7); in order to further demonstrate that the protection against 5FU is induced in chemoresistant cells exposed to 5FU, we treated chemoresistant colon carcinoma cell lines (HT-29 and HCT116p53KO) with 5FU and used such conditioned medium to protect HCT116 chemosensitive cells from 5FU-induced toxicity. Consistently, HCT116 showed an increased protection against 5FU action when treated with chemoresistant-cells-conditioned medium (Figure 8). Noteworthy, the main genetic difference between chemosensitive HCT116 and chemoresistant HCT116p53KO cell lines is the knocking out of *TP53* gene in the latter, and this raises the question on the possible role of p53 in the 5FU-induced TGF-β pathway activation, which would need further investigations.

In conclusion, this study demonstrated for the first time that the 5-fluorouracil treatment activated the TGF-β pathway in drug resistant colorectal carcinoma cells in *in vivo* and *in vitro* models. The specific abrogation of TGF-β pathway was able to restore sensitivity to chemotherapeutic action by specifically modulating the gene expression profile. The TGF-β pathway activation of 5FU-stimulated chemoresistant cancer cells conferred protection, by modulating surrounding microenvironment as well as cell mechanisms of death and proliferation genes, against 5FU toxicity.

## MATERIALS AND METHODS

### Reagents and antibodies

Lithium Chloride (LiCl), TGF-β1, Propidium Iodide, DNAse-free RNAse, Protease inhibitor cocktail, Phosphatase inhibitor cocktail #2, Phosphatase inhibitor cocktail #3 were purchased from Sigma Aldrich, SB431542 was purchased from Selleck Chemicals. Anti-TGF-βR1 (1:50, Rabbit polyclonal, Abcam), anti-SMAD3 (1:75, Rabbit monoclonal [EP568Y], Abcam), anti-SMAD4 (1:150, Rabbit polyclonal, Sigma Aldrich), anti TGF-β1 (1:100, Rabbit polyclonal, Sigma-Aldrich) primary antibodies were used for immunohistochemistry. Anti-Ki67 (1:100, Rabbit monoclonal [SP6], Abcam), anti-TGF-βR1 (1:50, Rabbit polyclonal, Abcam), anti-SMAD3 (1:300, Rabbit monoclonal [EP568Y], Abcam) primary antibodies were used for immunofluorescence analysis. HRP-conjugated anti-rabbit (ImmPRESS Reagent Kit, Vector Laboratories) or Alexa Fluor 488-conjugated donkey anti-rabbit (Life Technologies) secondary antibodies were used. For immunoblot analyses were used the following primary antibodies: anti-TGF-βR1 (Rabbit polyclonal, Cell Signaling Technologies), anti-SMAD3 (Rabbit monoclonal [EP568Y], Abcam), anti-p-S204-SMAD3 (Rabbit polyclonal, Abcam), anti-SMAD4 (Rabbit polyclonal, Sigma Aldrich), Anti GAPDH (Mouse Monoclonal, [GAPDH-71.1], Sigma-Aldrich). Secondary antibody used: ECL sheep anti-mouse IgG, Horseradish Peroxidase linked whole antibody (1:5000, Ge Healthcare), ECL donkey Anti-rabbit IgG Horseradish Peroxidase linked whole antibody (1:5000, Ge Healthcare).

### Xenograft model, immunohistochemistry and microvasculature density quantification

Xenograft model and treatments were performed as previously reported [19]. Briefly, HCT116p53KO chemoresistant cells were subcutaneously injected into the left flank of the mouse and HCT116 chemosensitive cells were injected into the contralateral flank, when the tumors reached the average volume of 100mm^3^, animals were randomized and distributed in the 4 treatment groups. After 21 days of treatment, animals were sacrificed and xenografted tumors collected. Resected tumors were put in formalin. Sections were then dehydrated, diafanized with xylene, put in paraffin, sectioned with microtome and put on slides. After deparaffinization, citrate based antigen retrieval was performed. Blocking was performed using Normal Horse Serum (ImmPRESS Reagent Kit, Vector Labs). Slides were then incubated with specific primary antibody diluted in PBS-Tween 0.1% for 1 hour in a humid chamber, avoiding drying of specimens. Slides were then incubated with HRP-conjugated anti-rabbit secondary antibody. For the detection of mouse microvasculature, after deparaffinization, slides were blocked with Carbo-Free™ Blocking Solution (Vector Laboratories) and incubated with Biotinylated GSL I –isolectin B4 (10 μg/ml, Vector Laboratories). VECTASTAIN^®^ ABC peroxidase was then applied to the specimens.

Processed slides were finally added with 3,3′-diaminobenzidine (DAB, Vector Laboratories) to develop the chromogenic reaction, and counter-stained with Hematoxylin (Vector Laboratories). Coverslips were mounted after de-hydration of the sections, using Permanent Mounting Medium (Vector Laboratories). Images were digitally acquired with ScanScope (Aperio) and quantified using ImageScope software (Aperio). The quantification was performed on the whole tumor section excluding necrotic areas and using Positive Pixels Count or Nuclear algorithm (Aperio), on the basis of the antigen localization.

### Cell lines, 3D cell culture and treatments

HCT116 and HCT116p53KO colon carcinoma cell lines were a kind gift of Dr. Bert Vogelstein (Johns Hopkins University, Baltimore, MD), HT-29 cells were from American Type Culture Collection (LGC Standards, Sesto San Giovanni, Italy). All cell lines were authenticated by STR analysis at Promega. Upon arrival, cells were expanded and frozen as a seed stocks of first or second passage. All cells were passaged for a maximum of 4 weeks, after which new seed stocks were thawed for experimental use. HCT116, HCT116p53KO and HT-29 cells were grown in McCoy's 5A-Glutamax medium with 10% FBS (Gibco, not Heat Inactivated), 100 U/ml Penicillin and 100 μg/ml Streptomycin. All cells were maintained in a 37°C incubator at 5% CO2.

For 3D culture, cells were seeded at a density of 1*10^5^/ml on a gelled bed of Extracellular Matrix (Cultrex^®^, Trevigen) in McCoy's 5A-Glutamax medium supplemented with 4% FBS (Gibco), 100 U/ml Penicillin and 100 μg/ml Streptomycin and 2% of Cultrex^®^, in order to create an Extracellular Matrix (ECM) gradient, modified from Debnath *et al* [45]. Three days after seeding, cells were pre-treated with LiCl (10mM) or with SB431542 (10μM) or TGF-β1 (10 ng/ml). After 24 hours of pre-treatment, old medium was discarded and fresh medium containing 5-Fluorouracil (5FU, 200 μM) and Lithium, SB431542 or TGF-β1 was added and maintained for 72 hours. For conditioned medium experiments, 3D-cultured HT-29 and HCT116p53KO cells were treated with vehicle or 5FU, and after 48h of conditioning, chemoresistant cells conditioned medium was used to treat 3D-cultured HCT116 cells for 72h. SB431542, when used, was added simultaneously to the conditioned medium.

### Immunofluorescence

For immunofluorescence analysis, cells were seeded in 8-well chamber slides (LabTek Chamber slides, Thermo Fisher Scientific), cultured and treated as described in the previous section. At the end of treatments cells were fixed with 4% Formaldehyde and, for intracellular antigens detection, permeabilized with Triton X-100 0.5% in PBS. Blocking with BSA 3% was performed to prevent non-specific binding of the antibodies. Cells were then incubated with primary antibody diluted in BSA 3% for 1 hour and for 30 minutes with appropriate secondary antibodies. Coverslips were mounted on slides using ProLong Gold mounting medium with DAPI (Life Technologies). Slides were imaged with a Zeiss Axioskope 2 microscope (Zeiss) equipped with fluorescence lamp and filters and a high-resolution digital camera (C4742–95, Hamamatsu Photonics). Single channel grey-scale images were quantified using ImageJ Software [46]. Threshold was fixed and applied to all images stained with the same antibody. For nuclear antigens, images were processed and threshold was fixed in order to measure only nuclear staining signal. Obtained fluorescence intensity measurements were normalized to DAPI fluorescence signal. The images were then processed with Adobe Photoshop CS6 software for color assignation to the corresponding fluorescence signal.

### Cell lysis, SDS-PAGE and western blotting

At the end of the treatments, cells were detached from gelled ECM using CellSperse™ (Trevigen) solution. Collected cells were lysed with a modified RIPA buffer: TrisHCl 50mM, NaCl 500mM, EDTA 1mM, EGTA 1mM, DTT 1mM, Protease inhibitor cocktail, Phosphatase inhibitor cocktail #2, Phosphatase inhibitor cocktail #3. Total protein extracts were quantified by Bradford assay (Sigma Aldrich), following manufacturer's instructions. Protein extracts were then processed to be loaded on NuPAGE Bis-Tris pre-casted mini gels (Life Technologies) following manufacturer instructions. Blotting onto nitrocellulose membrane (Life Technologies) was performed using iBlot System 2 (Life Technologies).

After blocking, membranes were incubated with the selected primary antibody and then with the appropriate secondary antibody. Super Signal West Dura Extended duration substrate (Thermo Scientific) was added to the membranes and chemiluminescent signal was digitally acquired by GBox (Syngene).

### Cell death analysis by propidium iodide incorporation assay

The cell death evaluation by propidium iodide incorporation of 3D-cultured cells was performed according to a modified version of the protocol from Riccardi *et al* [47]. Briefly, at the end of the treatments cells were detached from gelled ECM using CellSperse™ (Trevigen) solution. Cells were then fixed in 70% v/v ethanol at −20°C. DNA was extracted using a solution of Na_2_HPO_4_ and Triton X-100. Staining solution (Propidium Iodide 20μg/ml, DNAse free RNAse 200μg/ml) was then added to cell suspension and incubated on dark for 1h. Fluorescence was assessed using FACS Aria flow cytometer (Becton Dickinson). Data were analyzed using Cytobank software [48].

### RNA extraction, RT^2^ array and single gene validation

CellSperse™ solution was used to recover cells grown on Cultrex^®^, following manufacturer instructions. RNeasy^®^ Mini Kit (Qiagen) was used to extract RNA from cells grown on a thin layer of Cultrex^®^. RNA samples were treated with DNase to ensure elimination of genomic DNA. At the end of the procedure, the RNA concentration was measured with Nanodrop 2000 (Thermo Scientific) and samples were checked for RNA quality and integrity by 1.5% agarose gel electrophoresis in denaturing conditions. One microgram of extracted RNA was then converted to cDNA by using the RT^2^ First Strand Kit (Qiagen) according to manufacturer's instructions.

The expression of 84 Human TGF-β Signaling Targets genes was analyzed by RT^2^ profiler PCR array (PAHS-235ZA, Qiagen) using the StepOne Plus instrument (Applied Biosystems) following the manufacturer's protocol. Two independent experiments were performed for each group of treatment. Untreated cells were used as reference control sample. The mRNA expression levels of each gene in each cell treatment were normalized using the expression of the housekeeping genes *B2M*, *GAPDH*, *RPLP0*, *HPRT1* and *ACTB.* The results were confirmed by qRT-PCR experiments on selected genes by using StepOne Plus instrument (Applied Biosystems). At least three independent experiments were performed for each single gene validation. The primers used for qRT-PCR were selected from PrimerBank [49–51] and are listed in Supplementary Table S1. Data, normalized for *B2M* gene, are expressed as fold change value respect to the untreated cells according to the 2^−ΔΔCt^ algorithm. The array data discussed in this publication have been deposited in NCBI's Gene Expression Omnibus [52] and are accessible through GEO Series accession number GSE77927 (https://www.ncbi.nlm.nih.gov/geo/query/acc.cgi?acc=GSE77927).

### Statistical analysis

Data are presented as means ± SEM. Statistical analysis were performed with ANOVA test followed by Tukey's Test for multiple comparisons. Exact P values are indicated in figures or in legends and a P value <0.05 was considered as statistically significant. Statistical analysis was performed using GraphPad Prism (GraphPad Software).

## SUPPLEMENTARY MATERIALS FIGURES AND TABLE


