# Supplementary material for: The TGF-β pathway is activated by 5-fluorouracil treatment in drug resistant colorectal carcinoma cells
Source: Oncotarget. 2016 Mar 3;7(16):22077–91. doi: 10.18632/oncotarget.7895 (PMC5008345; doi:10.18632/oncotarget.7895)
Supplement: Supplementary file 1 [file oncotarget-07-22077-s001.pdf]

SUPPLEMENTARY MATERIALS

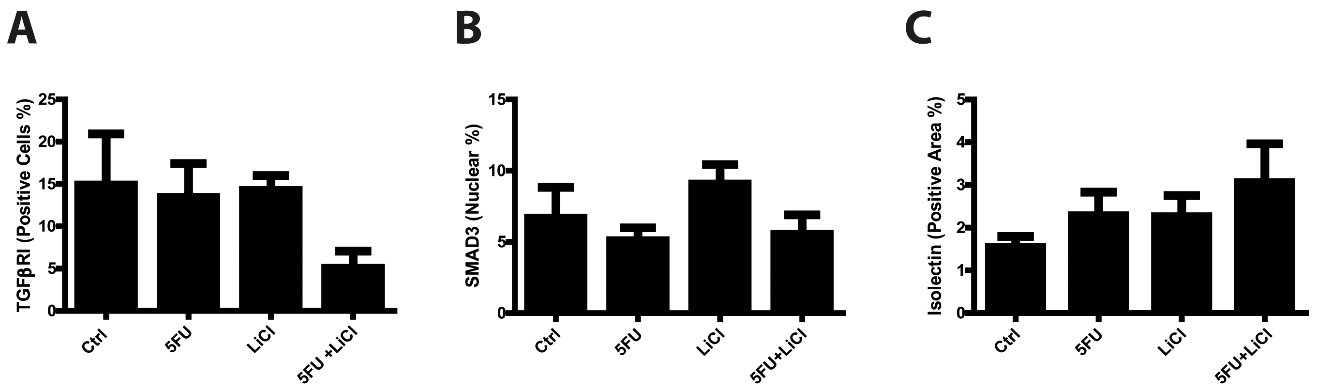

Supplementary Figure S1: 5-fluorouracil treatment did not cause any modulation of TGF-β pathway in xenografted chemosensitive cells. TGFβ pathway was not involved in HCT116 xenografted tumors drug response. Quantification was performed on whole tumor sections excluding necrotic areas (8 sections per group of treatment). No significant differences were detected among groups in terms of TGFβ-RI expression **A.**, SMAD3 sub-cellular localization **B.** nor vascularization **C.** Error bars represent SEM.

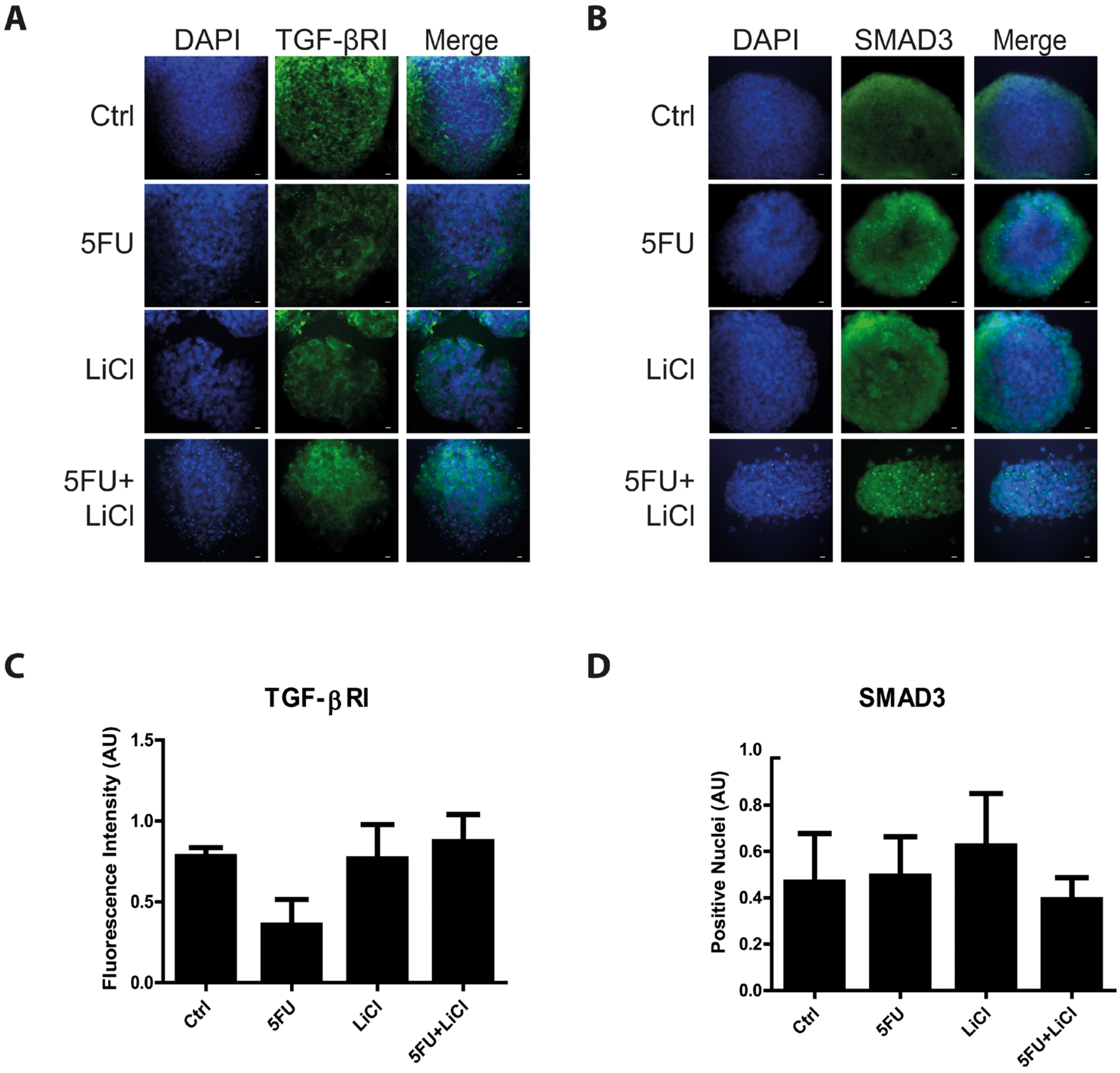

Supplementary Figure S2: 5-fluorouracil treatment did not cause any modulation of TGF-β pathway in 3D-cultured chemosensitive cancer cells. Representative pictures of immunofluorescence analysis in 3D-cultured chemosensitive HCT116 cancer cells. Cells were immunostained for TGF-βRI **A.** or SMAD3 **B.** (green) and with DAPI (blue). Bars represent 20 μm. Differently from what observed in chemoresistant cells, LiCl administration did not influence TGF-βRI expression **C.** in HCT116 cells in any treatment analyzed (control, 5FU, LiCl or 5FU+LiCl). **D.** SMAD3 (green) nuclear localization did not reveal any significant changes in consequence of the indicated treatments. No significant differences among groups were detected. Error bars represent SEM.

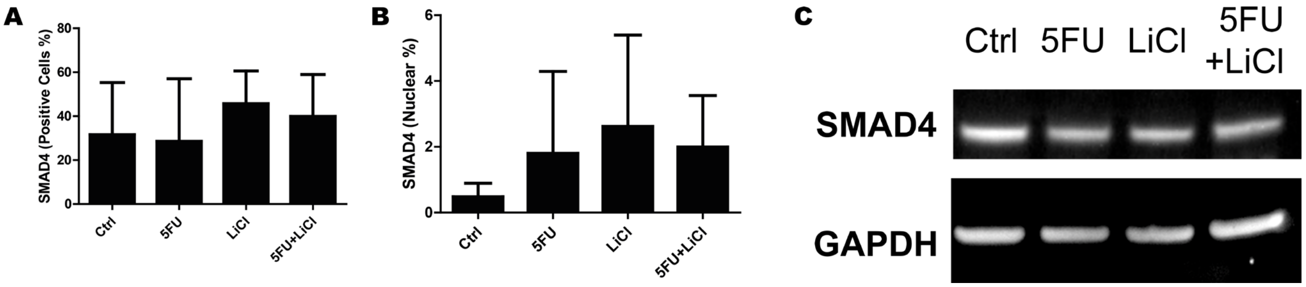

Supplementary Figure S3: SMAD4 expression and localization did not change among treatments *in vivo* and *in vitro*. Expression **A.** and nuclear localization **B.** of SMAD4, a co-SMAD acting with SMAD3 downstream the TGFβ pathway, were analyzed by IHC in sections of xenografted chemoresistant tumors and by immunoblotting in 3D-cultured tumor cells **C.** No significant changes were observed among treatments. Error bars represent SEM.

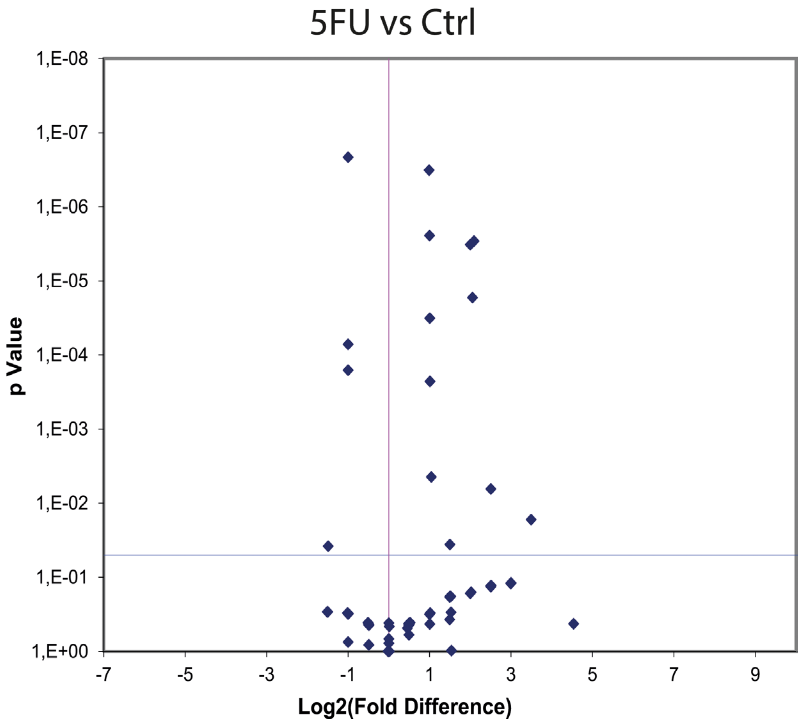

Supplementary Figure S4: 5FU modulates TGF-β target genes expression in chemoresistant colon carcinoma cells. Volcano plot of gene expression array (84 Human TGF-β Signaling Targets genes, RT^2 profiler PCR array PAHS-235ZA, Qiagen) on HCT116p53KO cells. The graph shows that 5FU treatment caused an increase of transcription of 52 out of 84 TGFβ target genes analyzed as compared to control cells, suggesting a specific response by chemoresistant cells to chemotherapeutic action. Genes are represented by points. In the X axis is plotted the Log_2 of fold change of genes in cells treated with 5FU normalized to control cells. In Y axis are plotted the *P*-values of the t-student test between the two groups of treatment. The horizontal line stands for the threshold *P*-value (P=0.05).

Supplementary Table S1: List of the primers used in qRT-PCR experiments

Gene	Forward Primer	Reverse Primer	PrimerBank ID
ACVRL1	5'-CGAGGGATGAACAGTCCTGG-3'	5'-GTCATGTCTGAGGCGATGAAG-3'	116734711c1
B2M	5'-GAGGCTATCCAGCGTACTCCA-3'	5'-CGGCAGGCATACTCATCTTTT-3'	37704380c1
BCL2L1	5'-GAGCTGGTGGTTGACTTTCTC-3'	5'-TCCATCTCCGATTCAGTCCCT-3'	20336333c1
FN1	5'-AGGAAGCCGAGGTTTTAACTG-3'	5'-AGGACGCTCATAAGTGTCACC-3'	47132556c2
ID1	5'-CTGCTCTACGACATGAACGG-3'	5'-GAAGGTCCCTGATGTAGTCGAT-3'	341865545c1
TGFB1	5'-CAATTCCTGGCGATACCTCAG-3'	5'-GCACAACTCCGGTGACATCAA-3'	260655621c3

qRT-PCR was carried out using 2X SYBR Green Master Mix (Applied Biosystem), primers with a final concentration of 0.1 µM and cDNA samples with a final concentration of 10 ng/µl in order to perform PCR reactions on 10 ng cDNA template. The PCR reactions were performed with the following thermal cycling conditions: 95°C for 10 min, followed by 40 cycles of 95°C for 15 sec and 60°C for 1 min. Melting curve analysis were performed to verify PCR specificity at the end of each PCR run.
